# Prevalence of potentially inappropriate medications and association with comorbidities in older adults with diabetes in an outpatient visitation setting

**DOI:** 10.3389/fpubh.2022.995948

**Published:** 2022-09-20

**Authors:** Lvliang Lu, Keqin Yao, Jiaqi Chen, Yujie Yang, Kai Wang, Jing Zheng, Pi Guo, Yunpeng Cai, Qingying Zhang

**Affiliations:** ^1^Department of Preventive Medicine, Shantou University Medical College, Shantou, China; ^2^Shenzhen Health Development Research and Data Management Center, Shenzhen, China; ^3^Shenzhen Institutes of Advanced Technology, Chinese Academy of Sciences, Shenzhen, China

**Keywords:** potentially inappropriate medication (PIM), polypharmacy, elder, diabetes, prevalence, comorbidity

## Abstract

**Aims:**

Potentially inappropriate medications had been found associated with adverse drug events such as falls, emergency department admissions and hospital readmissions. There is lack of information about the prevalence of potentially inappropriate medications and associated chronic conditions in older patients with diabetes in China. This study aimed to assess the prevalence of potentially inappropriate medications in older adults with diabetes in an outpatient visitation setting and the association with polypharmacy due to comorbidities.

**Materials and methods:**

This was a 3-year repeated cross-sectional study which conducted in outpatient setting of 52 hospitals in Shenzhen, China, using 2019 Beers criteria. The prevalence of potentially inappropriate medications, polypharmacy and comorbidities in older adults with diabetes in an outpatient setting was expressed as percentages. Logistic models were used to investigate the association between potentially inappropriate medication exposure and age, sex, polypharmacy and comorbidities.

**Results:**

Among the 28,484 older adults with diabetes in 2015, 31,757 in 2016 and 24,675 in 2017, the prevalence of potentially inappropriate medications was 43.2%, 44.88% and 42.40%, respectively. The top five potentially inappropriate medications were diuretics (20.56%), benzodiazepines (13.85%), androgens (13.18%), non-steroidal anti-inflammatory drugs (12.94%) and sulfonylureas (6.23%). After adjustment for age and polypharmacy, the probability of potentially inappropriate medication exposure was associated with chronic gastrointestinal diseases, followed by osteoarthritis and rheumatoid arthritis, chronic pulmonary disease, chronic kidney disease, tumor, dementia, chronic liver disease, hypertension, cardiovascular disease, cerebrovascular disease and hyperlipemia.

**Conclusion:**

Potentially inappropriate medications were common in older patients with diabetes in an outpatient visitation setting. Higher probability of potentially inappropriate medication exposure was associated with the comorbidity chronic gastrointestinal diseases as well as osteoarthritis and rheumatoid arthritis. To ensure that iatrogenic risks remain minimal for older adults with diabetes, the clinical comorbidities should be considered.

## Introduction

Potentially inappropriate medications (PIMs) among older adults are considered drugs that should be avoided because the risks to older adults outweigh the intended benefits for this population; drugs with insufficient evidence of their benefits when safer and equally or more effective medications are available; drugs that are used at irrational doses or duration; and drugs with high risk for drug–disease interactions (1). Several assessment tools have been compared in detecting PIMs (1–4). Determinants did not appear to significantly differ in accordance with the criteria used for classifying PIMs, except when criteria were limited to a subclass of drugs such as psychotropic drugs (5).

The Beers criteria have been the most frequently used to identify PIM use in older adults, ranging from 3.6 to 92.0% in health facilities and departments such as nursing homes, residential care facilities, inpatient departments and emergency departments of hospitals (5). A meta-analysis of observational papers published between 2002 and 2019 estimated the pooled prevalence of PIMs in people aged 65 or older in primary care at 33.3%, ranging from 35.9 to 59.2% in high-prevalence countries (United Kingdom, Belgium, Australia and New Zealand) and from 23.2 to 29.9% in low-prevalence countries (United States, Canada and The Netherlands) (6). Also, PIMs may account for 7.7% of hospital admissions (associated with older adults in primary care), 10.2% of adverse drug events, 15.0% of functional decline and 17.3% of emergency room visits; when PIM prevalence was 50% lower than present, these could be prevented (37 hospital admissions, 48 adverse drug events, 69 cases of functional decline and 79 emergency room visits per 1,000 events of each adverse outcome experienced by older patients in primary care) (6).

However, most studies have focused on quality enabling factors of PIM use, such as medical facilities at different levels, socioeconomic status, race/ethnicity, education, social supports, insurance and continuity of care, which cannot be easily altered, or inherently modifiable factors such as age, sex and geographic region. Many of these factors were not consistently significant in different healthcare systems (5, 7). Only the number of medications, which was strongly correlated with chronic conditions, physical and mental health and functional status, was the most consistent determinant of PIMs use (5). The association between number of drugs and PIMs used is probably confounded by the number and types of comorbidities (7, 8). Few studies have investigated the role of PIMs in polypharmacy due to comorbidities, which could be easily discussed or even reduced in medical practice (7). Additionally, most of these studies assessing PIMs prescribing was conducted outside China, with implications for particular healthcare systems.

Population aging and the increasing prevalence of diabetes are a healthcare challenge in China. The 7^th^ national population census in 2020 in China revealed that China had entered an aging society, with 190 million Chinese aged ≥ 65 years, accounting for 13.5% of total population (9). The latest research of the global and regional prevalence of diabetes in older adults (aged 65-99 years) conducted in 2019 indicated that about 35.5 million older adults have diabetes in China, representing one quarter of the world's diabetes patients and the highest prevalence in the world (10). A national cross-sectional study covering 31 provinces in mainland China in 2017 showed that the prevalence of diabetes was 28.8% in adults aged 60-69 years and 31.8% in those ≥ 70 years (11). Older adults with diabetes frequently have multiple chronic diseases, high risk of hypoglycemia and poor self-management ability, which requires specificity in prescription (12). Appropriate treatment for multi-comorbidities often requires multiple medications because clinicians are guided by clinical practice guidelines for each chronic condition (13–16). Miller et al. found that the risk of PIM exposure increased by 5.2% for each drug added to the list of medications for older adults (7). Adverse drug events in older adults caused by PIMs and polypharmacy may be associated with falls, fractures, constipation, emergency department admissions, hospital readmissions, and all-cause mortality (17–20), which have become a significant public health challenge.

Several surveys conducted in different health care settings showed that older adults with diabetes were the most commonly exposed to PIMs (5, 17, 21–23). Studies found that 24.9, 56.1, and 39.9% of older adults with diabetes had at least one PIM in The Netherlands, Canada and United States, respectively (22, 24, 25). A previous retrospective cohort analysis conducted in Beijing, which included 506,214 older adults in outpatient settings, found a prevalence of PIM of 38.07% (17), but knowledge of the prevalence of PIMs in older people with diabetes is lacking in China. Hence, studies of the association between PIMs and polypharmacy due to comorbidities in older adults with diabetes in outpatient settings are needed for reducing PIM exposure in clinical practice.

Because of the high prevalence of older adults with diabetes in China and high prevalence of PIMs in older adults with diabetes, more research is needed to evaluate the quality of medication use and assess the association between comorbidities and PIMs in Chinese older people with diabetes in outpatient settings. The two major questions of this study were to evaluate the prevalence of PIMs in older adults with diabetes in outpatient settings in China and to explore the association between PIMs and polypharmacy due to comorbidities in older adults with diabetes in outpatient visitation settings.

## Materials and methods

### Design

This repeated cross-sectional study was conducted according to the checklist for reporting results of internet E-survey guidelines and strengthening the reporting of observational studies in epidemiology (STROBE) (Supplementary Table 1).

### Participants and setting

This study involved using data for the years 2015, 2016 and 2017 from the Shenzhen Health Development Research and Data Management Center Database (SHDRDMCD) managed by the Shenzhen Health Development Research and Data Management Center. The SHDRDMCD includes detailed medical records for 52 hospitals outpatient departments in 2015, 2016 and 2017. Records for the outpatient visits covered patient identification, sex, date of birth, organization code, organization name, diagnosis and prescribed medications. Each drug was assigned a unique code based on its generic name. This database anonymizes patients by assigning a unique identification number to each patient. All data were checked and sorted in the Oracle database by professional platform administrators and medical workers under the supervision of the Shenzhen Municipal Health Commission to ensure the authenticity and validity of data. In accordance with article No. 32 of the Declaration of Helsinki formulated by the World Medical Association, the database was approved for research by the Review Committee of Shenzhen Institute of Advanced Technology, Chinese Academy of Sciences (No. SIAT-IRB-151115-H0084).

The inclusion criteria for the study were age 65 years or older before January 1 in 2015, 2016 or 2017; participants with type I or type II diabetes diagnosed by a clinician; one or more hospital outpatient visits in the 52 hospitals; and prescribed one or more medications. Exclusion criteria were participants prescribed only traditional Chinese medicine or Chinese patent medicine, which Beers criteria do not include, and lack of baseline information.

### Data collection

#### PIMs definition

We used the American Geriatrics Society 2019 Beers criteria (1) (the version based on updated evidence from the 2015 to 2017 literature review) to assess the PIMs for older adults with diabetes in an outpatient setting. The 2019 Beers criteria were applied by using a Delphi method by a 13-member group that included physicians, pharmacists and nurses who had participated in the 2015 update. The criteria are in five categories. Category I includes medications that should be avoided by most older adults; Category II, medications that should be avoided by older adults with certain conditions; Category III, medications that should be used with caution; Category IV, medications that should be avoided in combined treatment of older adults because of clinically important drug–drug interactions; and Category V, medications that should be dosed differently or avoided among older people with reduced kidney function.

The outpatient file of SHDRDMCD did not contain specific clinical characteristics for each patient such as laboratory test results and creatinine clearance, which are required in PIM assessment. In SHDRDMCD, the dosage was not uniformly recorded, which could be expressed in the weight of a drug or the number of dosage forms such as one capsule or one tablet. Also, there were no diagnoses such as syncope or delirium in outpatient files. Finally, the concurrent use of medication in older adults with diabetes were not included in the outpatient visitation record. Therefore, the assessment of PIM in the outpatient visitation setting for diabetes patients was mainly based on the category I, part of category II and category III of the 2019 Beers criteria list of medications that should be avoided in older adults. In addition, not all medications in the Beers criteria are available in China. Ultimately, we developed a list that contained 42 PIM items for the 2019 Beers criteria for PIM assessment in this study (Supplementary Table 2). The list contains the evaluated PIM items in this research and corresponding notes or reasons for exclusion. We identified patients' disease or syndromes, which are required in category II, in outpatient files by a joint description of the diagnoses and the International Classification of Disease, 10^th^ revision (ICD-10) codes (Supplementary Table 2).

#### Polypharmacy definition

We defined no polypharmacy as 0 to 4 chronically used drugs, moderate polypharmacy as 5–9 chronically used drugs, and severe polypharmacy as ≥ 10 chronically used drugs, the most frequently used polypharmacy definition method (26). Combined with Anatomical Therapeutic Chemical (ATC) codes, chronic use was defined as a drug prescribed for at least 90 days or at least once a month for each study year except for drugs prescribed for topical use, radiopharmaceuticals, surgical dressings, contrast media and general nutrients (ATC codes D, V, Y and Z) as well as records with invalid ATC codes. To calculate the number of different drugs for chronic use, we used the third level of the ATC, which describes pharmacological subgroups. Thus, one could count one chronically used drug with different substances within the same pharmacological subgroup, such as antiadrenergic agents.

#### Comorbidity definition

To explore the association between comorbidities and PIMs, we consulted the anatomical group and subgroup of therapeutics in ATC drug code categories to classify chronic comorbidities in older adults with diabetes. The clustering research conducted on multimorbidity among two million adults in China demonstrated that cerebrovascular disease, chronic kidney disease, hypertension, ischemic heart disease and osteoarthritis and rheumatoid arthritis were highly co-morbid with diabetes in older adults (27). This was in part why we choose below comorbidities to explore the correlation between comorbidities and PIMs in older adults with diabetes. Finally, cardiovascular disease, cerebrovascular disease, hypertension, hyperlipidemia, tumor, dementia, chronic liver disease, chronic pulmonary disease, chronic kidney disease, chronic gastrointestinal diseases and osteoarthritis and rheumatoid arthritis were investigated, and their ICD-10 codes are in Supplementary Table 3. For chronic conditions, the earliest recorded diagnosis code before each study year was accepted, except for tumor, with a diagnosis code recorded in the 5 years before each study year. For this study, multimorbidity was defined and classified as concurrently experiencing none, one, two, three and four or more chronic diseases besides diabetes. And comorbidity was presented as with or without specific disease listed above except for diabetes.

### Analysis

#### Outcomes

The primary outcome was the prevalence of PIM, polypharmacy and comorbidities in older adults with diabetes in outpatient settings per calendar year. The prevalence of PIM exposure was further classified into six levels based on the number of PIMs prescribed: 0 (reference, no PIM exposure), 1 (prescribed one PIM), 2 (two PIMs), 3 (three PIMs), 4 (four PIMs), and 5 (five or more PIMs). As what we setting above, the prevalence of polypharmacy exposure was classified into none, moderate and severe polypharmacy, multimorbidity exposure was classified into none, one, two, three and four or more chronic diseases besides diabetes, and comorbidity exposure was classified into with or without specific disease except for diabetes. The secondary outcome was to assess the association between polypharmacy due to comorbidities and the use of PIMs in older adults with diabetes.

#### Statistical analysis

The prevalence of PIMs, polypharmacy and comorbidities in older adults with diabetes in an outpatient setting was expressed as percentages with 95% confidence intervals (CIs) for each year investigated and was calculated by dividing the number of older adults with diabetes exposed to PIMs, polypharmacy and comorbidities (numerator) by the total number of older adults with diabetes included each year (denominator).

Unadjusted and adjusted logistic regression analyses were performed to assess the association of PIMs with study year (using 2015 as the reference year), age group (65-69 years as the reference age), sex, polypharmacy and comorbidities. The same method was used to assess the association of polypharmacy with study year, age group, sex, multimorbidity and comorbidity. A two-sided α = 0.05 was considered statistically significant. In addition, generalized variance-inflation factors (GVIFs) were calculated in adjusted logistic regression to explore the multicollinearity among variables analyzed. The function “Vif” in the R package “car” was used in the multicollinearity analysis (28). A GVIF value > 10 was considered strong multicollinearity in adjusted logistic regression. All analyses were performed with R 4.1.2 (R Development Core Team).

## Results

### Baseline characteristics and prevalence of multimorbidity

**Figure 1:**
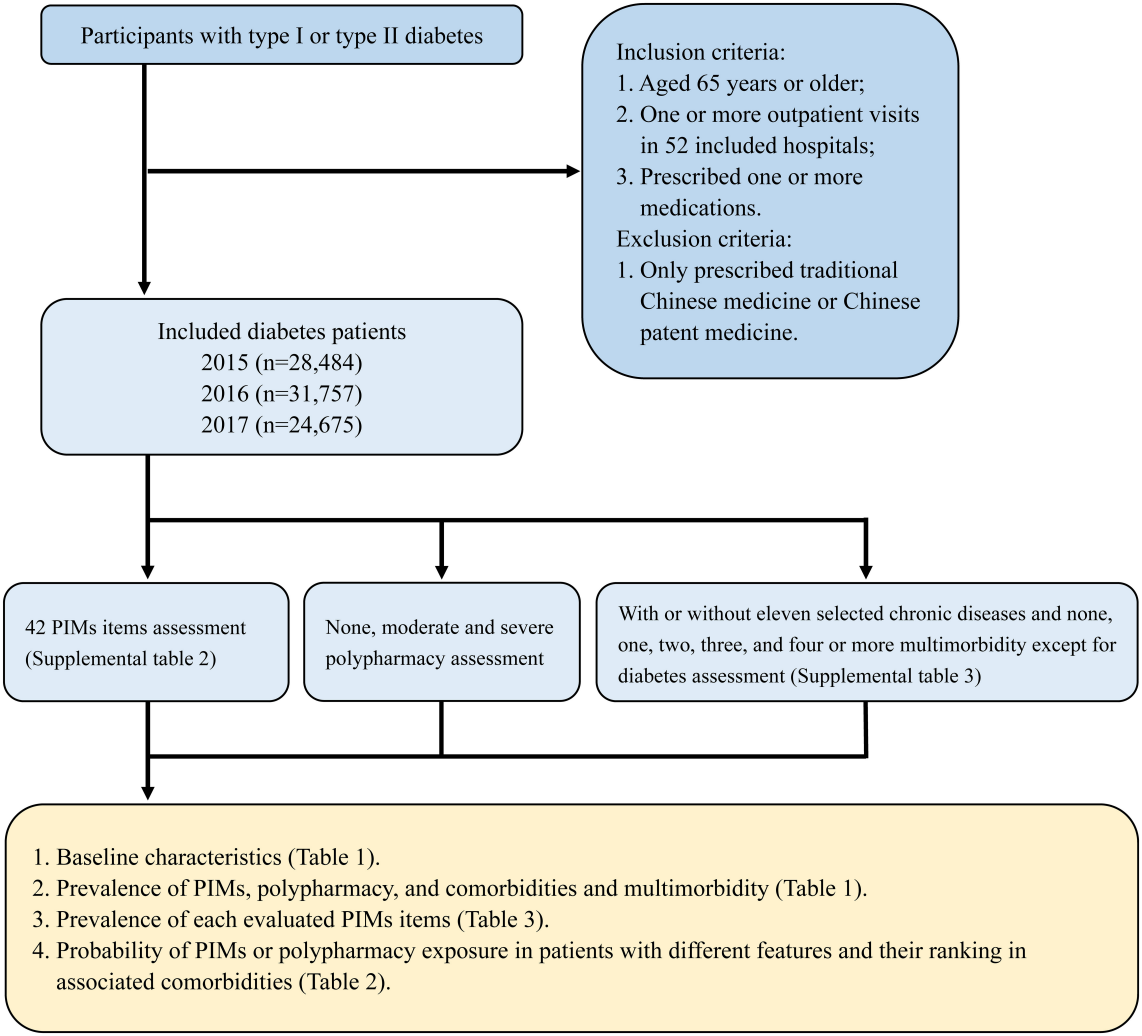
The flowchart of prevalence of potentially inappropriate medications (PIMs) and association with comorbidities in older adults with diabetes in an outpatient visitation setting which including the selection criteria of participant, investigating content, the main results and relevant tables.

The flowchart which including the selection criteria of participant, investigating content, the main results and relevant tables was shown in Figure 1. A total of 28,484 older adults with diabetes were detected in 2015, 31,757 in 2016 and 24,675 in 2017. The distribution of age and sex was similar among the years (Table 1). The top five chronic comorbidities in 2015, 2016 and 2017 were hypertension (59.78%, 70.09% and 74.50%, respectively), cardiovascular disease (35.50, 45.22, and 51.46%), hyperlipemia (29.03, 39.90, and 45.40%), osteoarthritis and rheumatoid arthritis (13.66, 21.14, and 23.25%) and cerebrovascular disease (12.06, 18.34, and 22.80%), and the ranking was consistent in the 3 years (Table 1). The prevalence of multimorbidity (≥ 1 comorbidity) in 2017 was 80.98% (95% CI 78.98–82.98), which was higher than in 2015 (76.43%, 95%CI 74.64–78.22) and 2016 (76.40, 95%CI 74.68–78.12%) (Table 1).

**Table 1:** Baseline characteristics of older adults with diabetes in 2015, 2016, and 2017.

	**2015 (*n =* 28,484)**		**2016 (*n =* 31,757)**		**2017 (*n =* 24,675)**		***P*-value**
	**n**	**%**	**n**	**%**	**n**	**%**	
**Age group, years**							0.062
65–69	10,135	35.58	11,762	37.04	9,068	36.75	
70–74	7,046	24.74	7,965	25.08	6,249	25.33	
75–79	5,770	20.26	6,247	19.67	4,809	19.49	
≥ 80	5,533	19.42	5,783	18.21	4,549	18.44	
**Sex**							0.128
Male	14,012	49.19	15,505	48.82	11,871	48.11	
Female	14,472	50.81	16,252	51.18	12,804	51.89	
**PIM**^†^ **group**							<0.001
0	16,180	56.80	17,506	55.12	14,213	57.60	
1	7,823	27.46	8,592	27.06	5,769	23.38	
2	2,752	9.66	3,238	10.20	2,602	10.55	
3	1,037	3.64	1,352	4.26	1,009	4.09	
4	428	1.50	596	1.88	520	2.11	
≥ 5	264	0.93	473	1.49	554	2.25	
**Polypharmacy**							<0.001
None (0–4)	13,802	48.46	14,707	46.31	11,253	45.60	
Moderate (5-9)	13,423	47.12	15,475	48.73	11,661	47.26	
Severe (≥ 10)	1,259	4.42	1,575	4.96	1,761	7.14	
**Comorbidity**							
Multimorbidity							<0.001
0	6,713	23.57	7,495	23.60	4,693	19.02	
1	7,031	24.68	6,271	19.75	5,027	20.37	
2	6,792	23.84	7,508	23.64	5,774	23.40	
3	4,920	17.27	5,844	18.40	5,405	21.90	
≥ 4	3,028	10.63	4,639	14.61	3,776	15.30	
Cardiovascular disease							<0.001
With	10,112	35.50	14,362	45.22	12,698	51.46	
Without	18,372	64.50	17,395	54.78	11,977	48.54	
Cerebrovascular disease							<0.001
With	3,436	12.06	5,824	18.34	5,626	22.80	
Without	25,048	87.94	25,933	81.66	18,938	76.75	
Hypertension							<0.001
With	17,027	59.78	22,257	70.09	18,383	74.50	
Without	11,457	40.22	9,500	29.91	6,292	25.50	
Hyperlipemia							<0.001
With	8,268	29.03	12,670	39.90	11,202	45.40	
Without	20,216	70.97	19,087	60.10	13,473	54.60	
Tumor							<0.001
With	829	2.91	1,525	4.80	1,552	6.29	
Without	27,655	97.09	30,232	95.20	23,123	93.71	
Dementia							<0.001
With	209	0.73	391	1.23	366	1.48	
Without	28,275	99.27	31,366	98.77	24,309	98.52	
Chronic liver disease							<0.001
With	1,075	3.77	1,710	5.38	1,640	6.65	
Without	27,409	96.23	30,047	94.62	23,035	93.35	
Chronic pulmonary disease							<0.001
With	2,177	7.64	3,743	11.79	3,333	13.51	
Without	26,307	92.36	28,014	88.21	21,342	86.49	
Chronic kidney disease							<0.001
With	1,577	5.54	2,486	7.83	2,362	9.57	
Without	26,907	94.46	29,271	92.17	22,313	90.43	
Chronic gastrointestinal disease							<0.001
With	193	0.68	523	1.65	535	2.17	
Without	28,291	99.32	31,234	98.35	24,140	97.83	
Osteoarthritis and rheumatoid arthritis							<0.001
With	3,890	13.66	6,712	21.14	5,737	23.25	
Without	24,594	86.34	25,045	78.86	19,049	77.20	

† PIM = potentially inappropriate medication.

### Prevalence of PIMs and the associated comorbidities

In older adults with diabetes, the percentage with at least one PIM exposure was 43.2% (95%CI: 41.86–44.53) in 2015, 44.88% (95%CI: 43.55–46.20) in 2016 and 42.40% (95%CI: 40.84–43.89) in 2017, for an absolute decrease of PIM exposure in 2017. For patients exposed to 4 or ≥ 5 PIMs, the PIM exposure was higher in 2016 and 2017 than 2015 (Table 1). The probability of PIMs was associated with the years 2016 and 2017 (OR = 1.02, 95%CI 1.01–1.03 and OR = 1.02, 95%CI 1.01–1.03) versus 2015 (Table 2). When we classified PIMs as different categories in the 2019 Beers criteria, older adults with diabetes and PIM exposure in the 3 years had similar rankings: 70.40% patients in 2015 were prescribed PIMs in category I, 69.30% in 2016 and 64.26% in 2017; followed by 26.91% patients in 2015 prescribed PIMs in category III, 28.01% in 2016 and 33.26% in 2017; and 1.63% patients in 2015 prescribed PIMs in category II, 2.70% in 2016 and 2.51% in 2017 (Table 3). When analyzed by PIM prevalence based on drug classes, the top five PIMs were diuretics (20.56%), benzodiazepines (13.85%), androgens (13.18%), non-steroidal anti-inflammatory drugs (NSAIDs) (12.94%) and sulfonylureas (6.23%). Besides, the specific PIMs identified in older adults with diabetes were shown in Table 3.

**Table 2:** Logistic regression analysis of factors associated with potentially inappropriate medications and polypharmacy.

**Covariate**	**Potentially inappropriate medications**							**Polypharmacy**						
	**Univariate analysis**			**Adjusted multiple logistics analysis**				**Univariate analysis**			**Adjusted multiple logistics analysis**			
	**OR^†^**	**95%CI^‡^**	***P***	**aOR^§^rank**	**aOR**	**95%CI**	***P***	**OR**	**95%CI**	***P***	**aOR rank**	**aOR**	**95%CI**	***P***
**Year of study**														
2015 (Reference)	1				–	–	–	1				–	–	–
2016	1.02	(1.01–1.03)	<0.001		–	–	–	1.03	(1.02–1.04)	<0.001		–	–	–
2017	1.02	(1.01–1.03)	<0.001		–	–	–	1.03	(1.02–1.04)	<0.001		–	–	–
**Age group, years**														
65–69 (Reference)	1						1			1				1
70–74	1.16	(1.12–1.19)	<0.001		1.03	(1.00–1.05)	0.03	1.13	(1.11–1.15)	<0.001		1.03	(1.01–1.04)	<0.001
75–79	1.34	(1.30–1.39)	<0.001		1.06	(1.03–1.09)	<0.001	1.27	(1.24–1.29)	<0.001		1.07	(1.05–1.09)	0.002
≥ 80	1.59	(1.53–1.64)	<0.001		1.09	(1.06–1.12)	<0.001	1.44	(1.40–1.47)	<0.001		1.12	(1.10–1.14)	<0.001
**Sex (Male/female)**	1.01	(0.98–1.03)	0.319		–	–	–	0.94	(0.93–0.96)	<0.001		0.98	(0.97–0.99)	0.001
**Polypharmacy**														
None (0–4 drugs)	1				1	–	–	–				–	–	–
Moderate (5–9 drugs)	2.38	(2.33–2.43)	<0.001		2.29	(2.19–2.39)	<0.001	–	–	–		–	–	–
Severe (≥ 10 drugs)	6.14	(6.01–6.28)	<0.001		5.56	(5.42–5.70)	<0.001	–	–	–		–	–	–
**Multimorbidity**														
0 (Reference)	1				–	–	–	1				–	–	–
1	1.45	(1.40–1.1.51)	<0.001		–	–	–	1.35	(1.32–1.38)	<0.001		–	–	–
2	2.00	(1.93–2.07)	<0.001		–	–	–	2.03	(1.99–2.08)	<0.001		–	–	–
3	2.76	(2.66–2.86)	<0.001		–	–	–	2.78	(2.72–2.85)	<0.001		–	–	–
≥ 4	4.22	(4.07–4.37)	<0.001		–	–	–	3.83	(3.74–3.91)	<0.001		–	–	–
**Comorbidity**														
Cardiovascular disease	2.03	(1.98–2.07)	<0.001	9	1.02	(1.00–1.04)	0.003	2.06	(2.03–2.09)	<0.001	1	1.53	(1.51–1.55)	<0.001
Cerebrovascular disease	1.64	(1.60–1.69)	<0.001	10	1.00	(0.98–1.03)	0.939	1.65	(1.62–1.68)	<0.001	7	1.25	(1.23–1.27)	<0.001
Hypertension	2.10	(2.05–2.15)	<0.001	8	1.04	(1.01–1.19)	<0.001	2.10	(2.07–2.14)	<0.001	2	1.49	(1.47–1.52)	<0.001
Hyperlipemia	1.64	(1.60–1.68)	<0.001	11	0.94	(0.92–0.96)	<0.001	1.82	(1.71–1.94)	<0.001	3	1.42	(1.35–1.50)	<0.001
Tumor	1.48	(1.41–1.56)	<0.001	5	1.16	(1.11–1.20)	<0.001	1.25	(1.21–1.30)	<0.001	11	1.11	(1.08–1.15)	<0.001
Dementia	1.99	(1.79–2.21)	<0.001	6	1.14	(1.05–1.24)	0.01	1.75	(1.62–1.88)	<0.001	5	1.27	(1.19–1.35)	<0.001
Chronic liver disease	1.46	(1.39–1.54)	<0.001	7	1.06	(1.04–1.08)	0.004	1.36	(1.31–1.41)	<0.001	8	1.15	(1.12–1.18)	<0.001
Chronic pulmonary disease	1.79	(1.73–1.86)	<0.001	3	1.18	(1.15–1.21)	<0.001	1.48	(1.45–1.52)	<0.001	9	1.14	(1.12–1.17)	<0.001
Chronic kidney disease	1.92	(1.84–2.01)	<0.001	4	1.17	(1.13–1.21)	<0.001	1.63	(1.59–1.68)	<0.001	6	1.25	(1.22–1.28)	<0.001
Chronic gastrointestinal diseases	2.65	(2.42–2.90)	<0.001	1	1.44	(1.34–1.54)	<0.001	1.79	(1.76–1.82)	<0.001	4	1.35	(1.33–1.37)	<0.001
Osteoarthritis and rheumatoid arthritis	1.93	(1.87–1.98)	<0.001	2	1.35	(1.32–1.38)	<0.001	1.45	(1.42–1.48)	<0.001	10	1.11	(1.09–1.13)	<0.001

† OR = odds ratio.

‡ CI = confidence interval.

§ aOR = adjusted odds ratio.

**Table 3:** Prevalence of PIMs by Beers category, drug class and medications of older adults with diabetes in 2015, 2016, and 2017.

**PIM^†^ category**	**Drug class**	**Medication**	**Prevalence**, ***n*** **(%)**		
			**2015**	**2016**	**2017**
**Category I: medications should be avoided by most older adults**			14,929 (70.40)	17,619 (69.30)	13,120 (64.26)
Anticholinergics	First-generation antihistamines	Chlorpheniramine	400 (1.91)	402 (1.58)	406 (1.99)
		Cyproheptadine	71 (0.34)	69 (0.27)	38 (0.19)
		Diphenhydramine	468 (2.24)	531 (2.09)	343 (1.68)
		Promethazine	347 (1.66)	467 (1.84)	204 (1.00)
		Triprolidine	46 (0.22)	24 (0.09)	9 (0.04)
	Antiparkinsonian agents	Trihexyphenidyl	49 (0.23)	72 (0.28)	44 (0.22)
	Antispasmodics	Atropine (excludes ophthalmic)	78 (0.37)	63 (0.25)	55 (0.27)
		Belladonna alkaloids	323 (1.55)	315 (1.24)	188 (0.92)
Antithrombotics		Dipyridamole	36 (0.17)	25 (0.10)	22 (0.11)
Anti-infectives		Nitrofurantoin	5 (0.02)	10 (0.04)	5 (0.02)
Cardiovascular	Peripheral alpha-1 blockers	Doxazosin	308 (1.47)	276 (1.09)	222 (1.09)
		Prazosin	55 (0.26)	60 (0.24)	55 (0.27)
		Terazosin	31 (0.15)	52 (0.20)	50 (0.25)
		Digoxin	297 (1.42)	385 (1.51)	411 (2.01)
		Nifedipine, immediate release	199 (0.95)	273 (1.07)	225 (1.10)
		Amiodarone	198 (0.95)	220 (0.87)	212 (1.04)
Central nervous system	Antidepressants	Amitriptyline	28 (0.13)	45 (0.18)	32 (0.16)
		Clomipramine	1 (0.001)	3 (0.01)	2 (0.01)
		Paroxetine	81 (0.39)	99 (0.39)	87 (0.43)
	Antipsychotics	Perphenazine	21 (0.10)	21 (0.08)	19 (0.09)
		Chlorpromazine	9 (0.04)	7 (0.03)	9 (0.04)
		Penfluridol	0 (0.00)	1 (0.001)	0 (0.00)
		Sulpiride	10 (0.05)	14 (0.06)	8 (0.04)
		Tiapride	8 (0.04)	7 (0.03)	3 (0.01)
		Haloperidol	16 (0.08)	11 (0.04)	8 (0.04)
		Amisulpride	0 (0.00)	5 (0.02)	3 (0.01)
		Aripiprazole	8 (0.04)	11 (0.04)	10 (0.05)
		Clozapine	24 (0.11)	29 (0.11)	21 (0.10)
		Olanzapine	159 (0.76)	249 (0.98)	216 (1.06)
		Quetiapine	46 (0.22)	84 (0.33)	69 (0.34)
		Risperidone	37 (0.18)	53 (0.21)	35 (0.17)
		Ziprasidone	0 (0.00)	4 (0.02)	3 (0.01)
	Barbiturates	Phenobarbital	58 (0.28)	53 (0.21)	25 (0.12)
	Benzodiazepines	Alprazolam	865 (4.14)	1,115 (4.39)	958 (4.70)
		Estazolam	1,596 (7.64)	1,735 (6.83)	1,593 (7.81)
		Lorazepam	35 (0.17)	46 (0.18)	28 (0.14)
		Oxazepam	19 (0.09)	36 (0.14)	22 (0.11)
		Clonazepam	185 (0.89)	237 (0.93)	200 (0.98)
		Diazepam	287 (0.37)	278 (1.09)	230 (1.13)
	Nonbenzodiazepine, benzodiazepine receptor agonist hypnotics	Eszopiclone	15 (0.07)	80 (0.31)	68 (0.33)
		Zolpidem	376 (1.80)	447 (1.76)	361 (1.77)
		Zaleplon	5 (0.02)	15 (0.06)	6 (0.03)
Endocrine	Androgens	Testosterone	12 (0.06)	16 (0.06)	14 (0.07)
		Desiccated thyroid	552 (2.64)	717 (2.82)	512 (2.51)
		Estrogens with or without progestins	1 (0.001)	3 (0.01)	0 (0.00)
		Growth hormone	1 (0.001)	0 (0.00)	0 (0.00)
		Insulin, sliding scale	2,104 (10.07)	2,715 (10.68)	2,111 (10.35)
		Megestrol	15 (0.07)	17 (0.07)	11 (0.05)
	Sulfonylureas, long acting	Glimepiride	1,349 (6.45)	1475 (5.80)	1,283 (6.29)
		Glyburide	23 (0.11)	14 (0.06)	11 (0.05)
Gastrointestinal		Metoclopramide	234 (1.12)	251 (0.99)	164 (0.80)
	Proton-pump inhibitors (≥ 8 weeks)	Omeprazole	0 (0.00)	1 (0.001)	1 (0.001)
		Rabeprazole	1 (0.001)	0 (0.00)	0 (0.00)
		Pantoprazole	0 (0.00)	1 (0.001)	2 (0.01)
		Lansoprazole	3 (0.01)	1 (0.001)	0 (0.00)
		Esomeprazole	0 (0.00)	1 (0.001)	0 (0.00)
Pain medications		Meperidine	107 (0.51)	153 (0.60)	105 (0.51)
	Non–cyclooxygenase-selective NSAIDs^‡^	Diclofenac	1,807 (8.65)	2,249 (8.85)	1,212 (5.94)
		Diflunisal	15 (0.07)	22 (0.09)	13 (0.06)
		Ibuprofen	780 (3.73)	796 (3.13)	419 (2.05)
		Ketoprofen	87 (0.42)	154 (0.61)	103 (0.50)
		Meloxicam	86 (0.41)	77 (0.30)	45 (0.22)
		Nabumetone	33 (0.16)	161 (0.63)	0 (0.00)
		Naproxen	1 (0.001)	0 (0.00)	0 (0.00)
		Oxaprozin	1 (0.001)	1 (0.001)	1 (0.001)
		Piroxicam	231 (1.11)	179 (0.70)	113 (0.55)
		Indomethacin	518 (2.48)	462 (1.82)	411 (2.01)
	Skeletal muscle relaxants	Chlorzoxazone	159 (0.76)	213 (0.84)	68 (0.33)
Genitourinary		Desmopressin	9 (0.04)	11 (0.04)	16 (0.08)
**Category II: PIMs that should be avoided by older adults due to drug-disease or drug-syndrome interactions**			346 (1.63)	689 (2.70)	513 (2.51)
Cardiovascular	Heart failure	Cilostazol	0 (0.00)	0 (0.00)	4 (0.02)
		NSAIDS	17 (0.08)	63 (0.25)	41 (0.20)
		COX-2^§^ inhibitors	3 (0.01)	8 (0.03)	5 (0.02)
		Thiazolidinediones (pioglitazone, rosiglitazone)	7 (0.03)	11 (0.04)	10 (0.05)
Central nervous system	Dementia or cognitive impairment	Anticholinergics	8 (0.04)	11 (0.04)	11 (0.05)
		Benzodiazepine	46 (0.22)	77 (0.30)	57 (0.28)
		Nonbenzodiazepine, benzodiazepine receptor agonist hypnotics	15 (0.07)	29 (0.11)	18 (0.09)
		Antipsychotics	45 (0.22)	75 (0.30)	64 (0.31)
	History of falls or fractures	Antiepileptics	24 (0.11)	39 (0.15)	24 (0.12)
		Antipsychotics	13 (0.06)	30 (0.12)	31 (0.15)
		Benzodiazepine	97 (0.46)	193 (0.76)	144 (0.71)
		Nonbenzodiazepine, benzodiazepine receptor agonist hypnotics	26 (0.12)	67 (0.26)	41 (0.20)
		TCAs	2 (0.01)	5 (0.02)	0 (0.00)
		SSRIs	15 (0.07)	39 (0.15)	28 (0.14)
		SNRIs	1 (0.001)	2 (0.01)	5 (0.02)
		Opioids	6 (0.03)	8 (0.03)	10 (0.05)
	Parkinson's disease	Antiemetics	7 (0.03)	15 (0.06)	4 (0.02)
		antipsychotics	14 (0.07)	17 (0.07)	16 (0.08)
**Category III: Medications to be used with caution in older adults**			5,626 (26.91)	7,107 (28.01)	6,770 (33.26)
		Dabigatran	15 (0.07)	24 (0.09)	44 (0.22)
		Rivaroxaban	12 (0.06)	29 (0.11)	43 (0.21)
		Antipsychotics	302 (1.44)	425 (1.67)	358 (1.75)
		Carbamazepine	127 (0.61)	133 (0.52)	81 (0.40)
	Diuretics	Spironolactone	814 (3.89)	1,025 (4.03)	1,154 (5.66)
		Indapamide	728 (3.48)	797 (3.14)	821 (4.02)
		Torasemide	89 (0.43)	82 (0.32)	120 (0.59)
		Furosemide	772 (3.69)	1,088 (4.28)	1,155 (5.66)
		Hydrochlorothiazide	1,657 (7.93)	1,743 (6.86)	1,713 (8.40)
		Tolvaptan	2 (0.01)	0 (0.00)	2 (0.01)
		Mirtazapine	56 (0.27)	92 (0.36)	60 (0.29)
		Oxcarbazepine	52 (0.25)	87 (0.34)	54 (0.26)
	SNRIs^¶^	Duloxetine	23 (0.11)	31 (0.12)	31 (0.15)
	SSRIs^††^	Citalopram	79 (0.38)	144 (0.57)	128 (0.63)
		Fluvoxamine	3 (0.01)	3 (0.01)	5 (0.02)
		Fluoxetine	146 (0.70)	168 (0.66)	119 (0.58)
		Paroxetine	81 (0.39)	99 (0.39)	87 (0.43)
		Sertraline	112 (0.54)	179 (0.70)	218 (1.07)
	TCAs^‡‡^	Amitriptyline	28 (0.13)	45 (0.18)	32 (0.16)
		Clomipramine	1 (0.001)	3 (0.01)	2 (0.01)
		Tramadol	269 (1.29)	322 (1.27)	204 (1.00)
		Dextromethorphan/quinidine	258 (1.23)	588 (2.31)	339 (1.66)

† PIM = potentially inappropriate medication.

‡ NSAIDs = non-steroidal anti-inflammatory drugs.

§ COX-2 = cyclooxygenase-2.

¶ SNRIs = serotonin-norepinephrine reuptake inhibitors.

†† SSRIs = selective serotonin reuptake inhibitors.

‡‡ TCAs = tricyclic antidepressants.

The probability of PIM was associated with older age; polypharmacy, especially severe polypharmacy (OR = 6.14, 95%CI 6.01–6.28); and ≥ 4 comorbidities (OR = 4.22, 95%CI 4.07–4.37). The GVIF value for multimorbidity was 21.826, which indicates strong multicollinearity among multi-morbidities and any comorbidity, so we eliminated it from the adjusted logistic regression model (Supplementary Tables 4, 5). Polypharmacy remained the most strongly associated variable (moderate polypharmacy: aOR = 2.29, 95%CI 2.19–2.39; severe polypharmacy: aOR = 5.56, 95%CI 5.42–5.70, Table 2). Probability of PIM exposure was associated with older age (age 70–74: aOR = 1.03, 95%CI 1.00–1.05; age 75–79: aOR = 1.06, 95%CI 1.03–1.09; age ≥ 80: aOR = 1.09, 95%CI 1.06–1.12, Table 2). As for comorbidity, instead of stating that older adults with diabetes and hyperlipemia had lower probability of PIMs, we would rather consider less PIMs related to hyperlipemia as compared with diabetes patients themselves who are exposed to insulin (sliding scale), glimepiride and glyburide, accounting for 16.69% of PIM exposure (Table 3). On adjusted logistic regression, the probability of PIM exposure was highest with chronic gastrointestinal diseases, followed by osteoarthritis and rheumatoid arthritis, chronic pulmonary disease, chronic kidney disease, tumor, dementia, chronic liver disease, hypertension, cardiovascular disease, cerebrovascular disease and hyperlipemia (Table 2).

### Prevalence of polypharmacy

Overall, the prevalence of polypharmacy (medication number > 4) was 51.54% (95%CI 50.73–52.36) in 2015, 53.69% (95%CI 52.90–54.48) in 2016 and 54.40% (95%CI 53.36–55.43) in 2017 (Table 1). In total, 4.42% (95%CI 4.18-4.66), 4.96% (95%CI 4.72–5.20) and 7.14% (95%CI 6.84–7.39) of older adults with diabetes were exposed to severe polypharmacy (medication number ≥ 10) in 2015, 2016 and 2017, respectively (Table 1). Furthermore, univariate analyses confirmed that the probability of polypharmacy was associated with the years 2016 and 2017 (OR = 1.03, 95%CI 1.02–1.04 and OR = 1.03, 95%CI 1.02–1.04) versus 2015.

To understand the association between comorbidities and PIMs more clearly, we analyzed the risk of polypharmacy in older adults with diabetes and different comorbidities. Similar to results of univariate analyses of PIMs, probability of polypharmacy was associated with older age, sex, multimorbidity and any comorbidity (Table 2). Probability of polypharmacy was lower for men than women (OR = 0.94, 95%CI 0.93–0.96) (Table 2). On eliminating multimorbidity (GVIF value = 21.766), the adjusted logistic model showed the probability of polypharmacy exposure associated with older age (age 70-74: aOR = 1.03, 95%CI 1.01–1.04; age 75-79: aOR = 1.07, 95%CI 1.05–1.09; age ≥ 80: aOR = 1.12, 95%CI 1.10–1.14) (Table 2, Supplementary Tables 4, 5). The prevalence of polypharmacy was associated with female sex (Table 2). Finally, on adjusted logistic analysis, the probability of polypharmacy was associated with cardiovascular disease, followed by hypertension, hyperlipemia, chronic gastrointestinal diseases, dementia, chronic kidney disease, cerebrovascular disease, chronic liver disease, chronic pulmonary disease, osteoarthritis and rheumatoid arthritis, and tumor (Table 2).

## Discussion

PIMs have been found associated with adverse health outcomes and prevalent in older adults with diabetes outside China (6, 22, 23). In light of the large number of older adults with diabetes in China and the complexity of chronic conditions in this population, we need to evaluate the prevalence and related clinical features of PIM exposure in China (11, 12). This study showed that the prevalence of exposure to at least one PIM in older adults with diabetes ranged from 42 to 45% in this 3-year repeated cross-sectional study conducted in outpatient departments of 52 hospitals in Shenzhen, China. The top three chronic conditions associated with the probability of PIM exposure were chronic gastrointestinal diseases, osteoarthritis and rheumatoid arthritis, and chronic pulmonary disease. The top three chronic conditions associated with the probability of polypharmacy were cardiovascular disease, hypertension and hyperlipemia.

Overall, such high PIM prevalence, over 40%, was a non-negligible issue in older adults with diabetes. First, the absolute decrease in PIM exposure in 2017 might be associated with continuing attention to PIMs and the 2017 publication of criteria of potentially inappropriate medications for older adults in China (4). Second, the prevalence of PIMs in this study significantly differed from results of a population-based study performed in Canada (56.1%) and a repeated cross-sectional study of outpatient prescriptions in The Netherlands (24.9%), both using 2015 Beers criteria (24, 25). However, the study conducted in The Netherlands evaluated only 24 PIM items and lacked clinical information such as specific medical information and clinical lab data. The study performed in Canada included PIM items that did not consider duration of medication, such as proton-pump inhibitors scheduled for > 8 weeks. These limitations might increase or decrease the estimation of the prevalence of PIMs. With the large sample size of this repeated cross-sectional study, precise medical records of outpatient SHDRDMCD files and 39 out of 42 items of the 2019 Beers criteria evaluated, the prevalence of PIMs, over 40%, in older adults with diabetes in outpatient visitation settings in Shenzhen, China, was assessed accurately. Therefore, this issue cannot be negligible in older adults with diabetes, regardless of the clinical practice in outpatient visitation settings or in nursing intervention in communities all over the world.

This study showed the highest probability of polypharmacy for older adults with diabetes and cardiovascular disease, followed by those with hypertension, hyperlipemia, chronic gastrointestinal diseases, dementia, chronic kidney disease, cerebrovascular disease, chronic liver disease, chronic pulmonary disease, osteoarthritis and rheumatoid arthritis, and tumor. A 5-year repeated cross-sectional study evaluating polypharmacy and PIMs in middle-aged and older people with diabetes revealed that the prevalence ranged from 36% in the 45-54 age group to 65% in the ≥ 65 age group, substantially higher than the prevalence in the general population (24). Of note, the absolute increase in polypharmacy was larger for patients aged 55-64 years than ≥ 65 years. The younger age group might have increased number of comorbidities, followed by an increase in prescribing medication. The older age group showed less increase in comorbidities along with less increase in number of medications (24). Additionally, the prevalence of polypharmacy seemed to increase by year (51.54% in 2015, 53.69% in 2016 and 54.40% in 2017). This increase may be associated with elevated prevalence of multimorbidity by year. Therefore, the association between polypharmacy and various chronic comorbidities in older adults with diabetes would help us better understand the features of medication prescribing in such adults. The findings of this study suggest considering the clinical comorbidities feature of older adults with diabetes in clinical practice and polypharmacy management.

The probability of PIM exposure differed with different comorbidities in outpatient visitation settings. The highest probability was with chronic gastrointestinal diseases, followed by osteoarthritis and rheumatoid arthritis, chronic pulmonary disease, chronic kidney disease, tumor, dementia, chronic liver disease, hypertension, cardiovascular disease, cerebrovascular disease and hyperlipemia. In considering prescriptions for disease, the highest probability of PIM exposure with chronic gastrointestinal disease might be explained by lack of diagnosis of gastroparesis when prescribing metoclopramide (1). A key factor of increased PIM exposure was the frequent use of NSAIDs and indomethacin in older adults with diabetes and osteoarthritis and rheumatoid arthritis. In addition, two of the top five frequently used PIMs were related to diabetes (insulin [sliding scale] of androgens and sulfonylureas), for a higher exposure of PIMs in older people with diabetes. This observation might explain why older adults with diabetes and hypertension, hyperlipemia, cardiovascular disease and cerebrovascular disease, which are frequently associated with diabetes, presented lower probability of PIM exposure. Meanwhile, diuretics were the most frequently used PIMs because of the high prevalence of hypertension, hyperlipemia, cardiovascular disease and cerebrovascular disease in people with diabetes. As well, chronic insomnia and anxiety are highly prevalent in people with diabetes (29, 30), which might explain why benzodiazepines were the second frequently used PIM in older adults with diabetes.

As compared with other chronic comorbidities, the presence of hypertension, hyperlipemia and cardiovascular disease was associated with high probability of polypharmacy but low probability of PIM exposure on adjusting for polypharmacy. Undoubtedly, the increased number of medications is associated with increased likelihood that at least 1 of the medications will be inappropriate (8). For example, Miller et al. found increased risk of PIMs by 5.2% for each drug added to the list of medications for older adults (7). Our results for polypharmacy certainly relate to PIM use, but the probability of PIM exposure differed by different comorbidities. As compared with low prevalence of chronic gastrointestinal disease (<3.0%) accompanied by metoclopramide prescription (nearly 1.0%), a high prevalence of hypertension, cardiovascular disease, cerebrovascular disease and hyperlipemia was associated with relatively lower percentage of PIM exposure (5.2% for cardiovascular prescription and 20.56% diuretics). Hence, the chronic conditions and corresponding PIM prescriptions we summarized are crucial to reduce PIM exposure in clinical practice or in nursing intervention in community-dwelling older adults with diabetes. Therefore, we should concentrate on the chronic comorbidity status of older adults with diabetes except for the polypharmacy in clinical practice and medication management.

### Strengths and limitations

The strengths of our study include the 3-year repeated cross-sectional study design with a large sample size of older adults with diabetes from the outpatient SHDRDMCD file, assessing PIM prescribing based on three (of five) PIM categories (39 of 42 items) of the 2019 Beers criteria with available information from SHDRDMCD, exploring the chronic comorbidity of older adults with diabetes with information on diagnosis, evaluating the prevalence of polypharmacy and PIMs in older adults with diabetes and showing the features and ranking of the probability of polypharmacy and PIM exposure in older adults with diabetes. To reduce the potential biases of the results in assessing the prevalence of polypharmacy, PIMs and chronic comorbidity, our study has the following limitations. First, categories IV and V of the 2019 Beers criteria were excluded and so the prevalence of PIMs might be underestimated because of the lack of laboratory data and the concurrent use of medication. Second, only chronically used drugs, defined as a drug prescribed for at least 90 days or at least once a month, were included for polypharmacy assessment because we could not ascertain the concurrent use condition of medication in patients. Third, the patient's exact comorbid disease status in each study year was replaced by the definition of chronic comorbidity, which was limited to the earliest recorded diagnosis code before each study year and tumor with a diagnosis code recorded in the 5 years before each study year, which might overestimate the number of chronic conditions of patients. Fourth, our adjusted model lacked adjustment for the frequency of interaction with the healthcare system, which was related to the pathway of “the number and types of comorbid illness–polypharmacy–PIM exposure”. Fifth, the 2019 Beers criteria requires personalized risk–benefit assessment; for example, the use of diuretics might be justified in an individual. However, because no monitoring information is available in SHDRDMCD, we could not evaluate whether the use of these drugs was appropriate for an individual, but we identified them as PIMs. This situation might overestimate the prevalence of PIM exposure. Finally, this study excluded older adults with diabetes who did not take any drugs or only took traditional Chinese medicines, which might lead to an overestimation of PIM prevalence.

In conclusion, PIMs and polypharmacy were common in older adults with diabetes in Shenzhen, China. The higher probability of PIM exposure was associated with chronic gastrointestinal diseases and osteoarthritis and rheumatoid arthritis in addition to diabetes. The top five PIMs in older adults with diabetes were diuretics, benzodiazepines, androgens, NSAIDs, sulfonylureas, metoclopramide and indomethacin. Considering the associated adverse health outcome of PIMs, maintaining minimal iatrogenic risks for older adults with diabetes who are already vulnerable to these outcomes seems important. The clinical comorbidity features of such patients should be focused on apart from their polypharmacy status. Also, the rationality of prescribing diuretics, benzodiazepines, androgens, NSAIDs, sulfonylureas, metoclopramide and indomethacin in such patients should be considered in outpatient visitation settings.

## Data availability statement

The original contributions presented in the study are included in the article/Supplementary material, further inquiries can be directed to the corresponding author.

## Author contributions

KY, YY, JZ, and YC: database management, acquisition of data, and research coordinator. LL, JC, KW, PG, and QZ: conception, research design, and analysis and interpretation of data. LL and KY: manuscript draft. QZ and YC: manuscript revise. All authors contributed in the critical review of the manuscript and approved its submitted version.

## Funding

This study was supported by Natural Science Foundation of Guangdong Province, Grant No.: 2021A1515011193, the Strategic Priority Research Program of Chinese Academy of Sciences, Grant No.: XDB 38050100, Shenzhen-Hongkong Join Research Funding, Grant No.: SGDX20201103095603009, and National Nature Science Foundation of China, Grant No.: NSFC 61902387.

## Conflict of interest

The authors declare that the research was conducted in the absence of any commercial or financial relationships that could be construed as a potential conflict of interest.

## Publisher's note

All claims expressed in this article are solely those of the authors and do not necessarily represent those of their affiliated organizations, or those of the publisher, the editors and the reviewers. Any product that may be evaluated in this article, or claim that may be made by its manufacturer, is not guaranteed or endorsed by the publisher.
